# Household Syphilis (Syphilis Œconomica), with Observations on Acquired Syphilis in Infants

**Published:** 1911-12

**Authors:** J. A. Nixon

**Affiliations:** Physician to the Bristol Royal Infirmary


### HOUSEHOLD SYPHILIS (SYPHILIS ŒCONOMICA), WITH OBSERVATIONS ON ACQUIRED SYPHILIS IN INFANTS.

BY

J. A. NIXON, B.A., M.B. Cantab., F.R.C.P.,
*Physician to the Bristol Royal Infirmary.*

---

THE transmission of syphilis by other means than sexual intercourse is probably commoner than we are wont to suppose. We always receive the protestations of syphilitic adults that coitus has not been the source of infection with a scepticism which worldly wisdom goes far to justify. But every now and again a case of acquired syphilis in a child raises a doubt whether we do not underestimate the frequency of the spread of syphilis by domestic and accidental as opposed to venereal contagion.

I have lately met with some instances of family spread of syphilis which have impressed upon me this important aspect of the disease. In the face of public apathy and ignorance of the transmissibility of so terrible an infection, it is as well to remind ourselves and the public that the great-pox may be communicated in the household from one member to another, if less frequently, not less innocently than the small-pox. We are inclined to forget Colles' view[1]: "The readiness with which this disease is communicated by contact cannot be exceeded in this property by any other disease with which I am acquainted. I look upon it as equally infectious with the itch itself. Another manner in which the disease is made to spread through the family is by the use of the same spoon, and drinking out of the same vessel with another of the family to whose mouth the disease may have spread. Those who are acquainted with the very scanty furniture of an Irish cabin will readily comprehend with what facility and rapidity the disease can be propagated in this manner."

In the transmission of domestic syphilis children play an important part. Heredo-syphilis is essentially contagious, and to belittle this is a dangerous doctrine ; and, further, we must not forget that acquired syphilis is by no means rare in infants. Between these two sources of contagion the family into which syphilis makes an entrance is in greater danger of an epidemic than is generally supposed. All the older writers recognised this, but in later authors the matter meets with less weighty consideration, and is often dismissed in very summary fashion.

Diday,[2] in his book on *Syphilis in New-born Children,* is no less insistent than Colles on the subject. He speaks of the extension of this family transmission assuming the proportions of a veritable epidemic.

Paré's[3] case is worth relating as one "of these concatenations of infections." "A certaine very good citizen of this citye of Paris granted to his wife, being a very chaste woman that conditionally shee should nurse her owne child of which she lately was delivered shee should have a nurse in the house to ease her of some part of her labour. By ill hap the nurse they took was troubled with this disease wherefore she presently infected the childe, the childe the mother the mother her husband and hee two of his children who frequently accompanied him at bed and board, being ignorant of that malignity wherewith he was inwardly tainted. . . . I had them in cure and by God's helpe healed them all, except the sucking childe which died in the cure. But the hired nurse was soundly lashed in the prison and should have been whipped through all the streets of the citie but that the magistrate had a care to preserve the credit of the unfortunate family."

Diday himself met with several cases where an infant got or gave the syphilitic infection ; but Facen's[4] case must suffice to illustrate the contrary process, where a heredo-syphilitic infant infected seven persons, the wet-nurse, a second child suckled by the wet-nurse, the mother of the second child, a girl of eighteen who had care of this latter child, a foster-child of the mother and this child's sister—a girl ten years of age—and lastly the mother of these two children, a woman of 50.

It is evident how in this instance children form the necessary channels of communication from one family to another. Adults are more liable to acquire syphilis accidentally or extra-genitally from children whom they fondle and kiss, than from other adults with whom their relations are less intimate. Lancereaux regarded the question as of serious import.

Paracelsus [5] pointed out the various modes of syphilitic contagion: "Infectio triplici via, videlicet coitu, partu, tactu."

Charles Musitan [6] relates the history of the nuns of Sorrento, who contracted syphilis from kissing a little girl who had been suckled by a syphilitic woman.

Sydenham [7] remarks: "Infants may take it from adults by the mere contact of the skin, as when they lie naked in the same bed with diseased persons." And he adds less correctly: "Adults, indeed, would not take it under such circumstances without impure coitus. Infants, however, having laxer flesh and finer skin, readily take the poison."

In former times and in other countries, where wet-nursing was more in vogue than in England, suckling was an important means of spreading syphilis. Ricordi, [8] in 1863 at Cazorezzi, described a foundling affected with syphilis who caused the infection of twenty-three individuals. In the same year at Ubolda a foundling equally suffering from hereditary syphilis transmitted the disease to eighteen individuals. A third epidemic at Marcalle in 1864 proved fatal in sixteen cases.

Portal [8] relates that a kind of scrofulous epidemic prevailed at Montmorency amongst the children at nurse. The evil became so great there that the Government sent Moraud and Lassonne, who ascertained that most of the children were syphilitic.

But few epidemics of this nature have been more striking than that described recently by J. F. Schamberg, [9] where a single evening's social entertainment was followed by "eight cases of lip-chancre, all transmitted from one individual by kissing games."

There is no need to multiply such quotations. All syphilologists agree as to the transmissibility of syphilis in the family and the household. We are sometimes inclined to regard the accident as so rare that it need not be considered as a practical possibility. Many of us have never seen an instance. Should we see it more frequently if we inquired more assiduously ? Lately several families have come under my notice in which syphilis spread by domestic means of infection.

**Case 1.**—In 1907 a girl, E. B., aged 15, attended at the Bristol Royal Infirmary suffering from a chancre in the centre of the dorsum of her tongue, with mucous tubercles on the tonsils, shotty glands in the neck, a sentinel bubo under the chin, and later a faint roseolous rash. Her mother had been under the care of Dr. Kenneth Wills at the Bristol General Hospital four months previously on account of secondary syphilis, which she admitted had been acquired by intercourse with a man other than her husband. At the time when the daughter came under my observation I found the father, a man of 62, an in-patient at the Eye Hospital with recent secondary syphilitic iritis. There was no evidence showing where the primary sore had been, and there was no scar suggesting that there had been a genital chancre.

**Case 2.**—Early in 1907 a married woman, Mrs. K., who had previously had one healthy child (Ivy K., aged 2 years), was deserted by her husband who had recently to his wife's knowledge acquired syphilis from a prostitute. Mrs. K. was not aware that he had transmitted the disease to her ; but presently, finding herself pregnant and deserted by her husband, she obtained admission to the Union Infirmary, and was delivered of a heredo-syphilitic baby that died soon after birth. Mrs. K. left the Infirmary and went to live with her married sister, Mrs. B., taking with her Ivy K., her daughter, aged 2 years.

Within two months Ivy K. came under observation at the Bristol Royal Infirmary with acquired syphilis, sore throat, roseolous rash, and condylomata. The site of the primary chancre was not discovered. Two months later Mrs. B.'s infant, aged 8 months, acquired a chancre on his lower lip, and transmitted the infection to his mother's breast, where a primary sore on the nipple developed. Both Mrs. B. and her baby persevered with treatment, but suffered from severe secondaries, and later from gummata. I have lost sight of Mrs. K. and her daughter Ivy.

**Case 3.**—In 1907 Dr. Stack showed me a patient named Emily B. (a married woman) with a chancre of the lower lip. Her mother was also under treatment at that time for secondary syphilis, the remains of a chancre of the tongue being still perceptible. Eighteen months later a sister of the first-named, a girl of 18 unmarried, came under my care with a chancre of the left tonsil.

**Case 4.**—In 1907 I attended a girl of 18 with roseola and condylomata, who denied genital infection and had an intact hymen. I do not know where the primary chancre had been, but at the same time her father was under my care being treated for gonorrhœa and syphilis of recent acquisition, antedating his daughter's infection.

**Case 5.**—In 1910 a girl of 14 came under observation for gummata of the legs. She had no signs of heredo-syphilis, but had a well-authenticated history of a chancre of the cheek at the age of four, acquired from her mother, who being at the time a ward maid in the Lock Ward of a Union Infirmary in London, had a little while before developed a chancre on one of her fingers.

**Case 6.**—Early in 1911 Dr. Watson-Williams transferred to my care a girl aged four who had been brought to his throat department at the Bristol Royal Infirmary with well-marked secondary syphilis, a ragged infiltrated ulcer of the left tonsil, and a sentinel bubo under the jaw. The source of her infection remained unexplained.

I have not here quoted all the cases of extra-genital infection which have come under my notice, but have confined myself to those examples in which the infection has been familial or domestic. Numbers 4 and 6 in the list are doubtful instances, but the remaining four show clearly the dangers of syphilis spreading in the home by non-venereal contagion. This is what has been called *syphilis œconomica*, or syphilis of the household, and the term is not identical with *syphilis insontium*; for surely no person acquires syphilis more innocently than the newly-wedded bride, whose husband proceeds, in the words of Kehoe,[10] "to blast the life of a good woman by polluting her with the rotten fruits of his licentious career." Nor does it include all cases of extra-genital infection, for this may be acquired by sexual intercourse. It is that variety of syphilitic infection which in Scotland long ago received the name of *sibbens*, and puzzled medical authorities by its obvious resemblance to syphilis, though "they could not bring themselves to acknowledge identity, because sibbens was not venereal."[11] The converse point of view, that syphilis spreads by venereal infection only, is at least acquiesced in by the medical profession at the present day, and is universal among the non-medical public. Hence it comes to pass that many cases of non-venereal syphilis are overlooked, especially among infants. It is doubtful whether we fully realise the ravages the disease is capable of in the midst of an unsuspecting and innocent family. Zarubin[12] holds strongly to the opinion that non-venereal syphilis is very common in Russia, and goes so far as to say "that it does not correspond with my observations to consider acquired syphilis in children is rarer than heredo-syphilis, and Kalmanowski, studying the subject in rural populations, agrees with me." Khijine found in rural districts of Russia extra-genital infection in 81 per cent. of all cases, and Militchevitch states that half the cases in Servia are extra-genital infection. Zarubin's conclusions are very interesting.

The non-venereal contagion is commoner in the country than in the towns.

Children are more liable to extra-genital infection than adults.

Infection by the mouth is the most frequent form of extra-genital syphilis.

Chancre of the pharynx is not less frequent than chancre of the lip.

The upper classes are as liable to non-venereal infection as the lower.

Children play an important part in the domestic spread of syphilis, both on account of the extreme infectivity of heredo-syphilis in infants, and by reason of their liability to acquire syphilis extra-genitally.

It should not be a very difficult matter to differentiate between acquired and inherited syphilis in infants, but the following points may help in the recognition of acquired syphilis.

The chancre is usually small, almost always extra-genital, and frequently overlooked or mistaken for some non-syphilitic affection.

Of course, it is not necessarily extra-genital. The instances of chancre of the prepuce, acquired by infants during the operation of circumcision, where sucking of the bleeding organ was resorted to for the arrest of hemorrhage by an infected operator, will be familiar to everyone ; and this may occur not only in male but in female children, as Bertin[13] records : "A girl, four months old, became the subject of a chancre on the upper and inner surface of the left labium. It was discovered that an aunt of this child, affected with syphilis, tended it and kissed it, sometimes gave it the breast to quiet it, and lastly that she washed its genital organs with water which she had previously put into her mouth to warm it."

Infection by the mouth is the most frequent form of extra-genital syphilis. In babies, kissing and the revolting practice of moistening the comforter or rubber teat of the bottle in the nurse's mouth lead to the majority of chancres being situated on the face, lips, or in the mouth.

The tongue and tonsils are not uncommon sites of extra-genital chancre. The tonsil is, perhaps, for all ages the commonest. Probably chancre of the tonsil or pharynx is conveyed thither by infected spoons or forks, and young children unaccustomed to and unhandy with these implements may often abrade the posterior parts of the mouth in greedy attempts to get their food safely swallowed.

Chancre of the conjunctiva is sometimes acquired by the practice of licking the eye to remove a foreign body. Nose, fingers, abdomen, scalp, and in fact any part of the exposed skin or vulnerable mucous membrane may be the seat of inoculation.

Vaccination chancre is more often talked of than seen ; and although Cory's[14] experiments showed that syphilis may be communicated in the vaccine lymph it is as well to bear in mind another possibility, illustrated by Gottheil's[15] family epidemic, which attacked all the members of a family of ten. "The disease was brought into the family by the eldest child, a girl aged 14 years, who contracted it in the ordinary way. She infected the vaccination wound of the youngest child in the family, a boy of two years, still nursing. This infant gave his mother a chancre of the nipple, and so in the course of a year the disease spread through the entire family, the father, strange to say, being the last to acquire it."

The chancre is always difficult to diagnose; experience teaches some of the peculiarities which it may present. First and foremost it is not necessarily or even usually indurated if it occurs on the skin; it will almost always be mistaken for a simple infection of some other sort. But it will prove very refractory to all treatment except mercury; it will tend to remain localised and circumscribed, and this may be of assistance by contrast with suppurating foci in infants, which spread and multiply as a rule very readily. There may be, if on or near a mucous membrane, an extraordinary degree of localised swelling. A chancre of the lip, once seen, forms a clinical picture which on the next occasion will be recognised across the room. There is very little pus discharging from an angry raw surface. If the chancre be on the skin it has a characteristic raspberry appearance; if near the nail it resembles a chronic whitlow; if on the face or scalp it may simulate impetigo, save that it is solitary, and, unlike impetigo, remains so; if on the lip or tongue it has the angry tumidity of an insect sting; if on the tonsil, in my experience it has usually been mistaken for diphtheria, for over a greatly swollen tonsil, which looks as if it were bulging with pus, there spreads a queer glairy whitish yellow, sometimes cartilaginous-looking film, which is not removable like a true membrane, nor formed by a coalescence of plugged follicles. The tonsil may be incised for quinsy—I have seen it done—and the exudation promptly coats the sides of the incision. Speaking generally, in infants a chancre will probably at first, at any rate, be missed. The secondaries are often benign, far less severe than in heredo-syphilis: a faint roseola, a morbilliform eruption, or a moist wash-leather seborrhœic rash are the usual varieties met with; or it may look no more than an intertrigo, where the roseola elsewhere is insignificant, but the moist fold of skin being chafed renders it more apparent.

It is a good plan to view a doubtful roseola through a piece of blue glass, whereby it is rendered easily visible. But condylomata are almost always present, and intertrigo, however severe from non-syphilitic causes, never produces anything dimly resembling condylomata. These condylomata are situated round the anus and vulva, and are practically invariably accompanied by mucous tubercles in the mouth, or snail crawls on the tonsils, or a characteristic chapping of the angles of the mouth. Very often the whole face looks puffy and swollen, suggesting acute nephritis. Periostitis may occur early, but as a rule comes later. There is invariably glandular enlargement in the neighbourhood of the primary sore. This takes the form of an indurated sentinel bubo. There may be considerable enlargement of other adjacent glands, with shotty swelling of all glands, and especially the posterior cervical chains. Anæmia is a prominent symptom, but in the cases I have seen there has not been any marked wasting. The marasmus of heredo-syphilis is absent. In other respects the disease runs the ordinary variable course of syphilis, passing on to tertiary manifestations of every kind.

The differential diagnosis between acquired and inherited syphilis in infants depends upon :—

(1) Finding the chancre. " A primary chancre existing in a child, and which has not commenced in it before the fifteenth day after its birth, is a valuable indication for arriving at the source of the disease. It exonerates the parents " (Diday, 199).

(2) The general appearance of the child is more healthy in the acquired form.

(3) The characteristic lesions of heredo-syphilis are absent in acquired syphilis, namely " natiform skull, epiphysitis, snuffles, bullæ of palms and soles, and in plaques of face and buttocks."

(4) There may be an incompatibility between the age of the patient and the quality of the eruption (*e.g.* roseola and condylomata at twelve years must depend upon acquired infection).

The tertiary lesions are impossible to distinguish, but it may help if one bears in mind that interstitial keratitis and deafness may be acquired, Hutchinson's teeth never.

The sunken bridge of the nose, if present, is positive evidence of heredo-syphilis, for snuffles does not occur in acquired syphilis. Many heredo-syphilitics escape this stigma, therefore its absence argues nothing. Early fundus changes are of the greatest value, disseminated choroiditis is essentially syphilitic in origin, and may in hereditary cases be present at birth. A well-marked case with old-standing choroidal atrophy is unmistakable, and cannot be associated with recently-acquired secondary syphilides. Myopic choroiditis must not be mistaken for syphilitic ; the refraction will help, and a myopic crescent or staphyloma is conclusive evidence that syphilis is not the sole cause of the fundus changes.

The hoarse cry is not characteristic of inherited syphilis, neither are condylomata, mucous tubercles, and fissures round the mouth. The relatively late appearance of lesions about the mouth in congenital syphilis is an important feature in contrast to the acquired form, where the earliest lesions are so often in the mouth. "Bardinet has rendered a true service to science and to practice in showing that most frequently congenital symptoms commence in the new-born child at the genitals or or anus, and do not attack the mouth until later." Dystrophic lesions are the mark of the heredo-syphilitic. The wizened, old, monkey-like child, the hare-lip, cleft palate, monstrosities and maldevelopment of various organs, whether partial or general, will distinguish inherited from acquired syphilis. Another inquiry of the utmost value is the investigation of the mother's history of pregnancy and parturition. Any woman may be assumed to have had syphilis who gives a history exemplifying Diday's law of decrease of the fœticidal action of the syphilitic poison.

It is from the recognition of acquired syphilis in children that the spread of syphilis in the family is most commonly detected. As a rule adults are rightly or wrongly assumed to have been infected by venereal contact, and some practitioners regard acquired syphilis in children as being commonly the result of a criminal assault. *Syphilis œconomica* is the true explanation of no small proportion of cases occurring in children, and extra-genital chancres about the mouth in adults, as well as in children, are more often than not due to infection from food utensils; especially is this the case when the primary sore is situated on the tongue or tonsil.

Gaucher and Flurin,[16] in their excellent study upon the situation of chancres in children under fifteen years of age, show from their own observations and those of Professor Fournier that the lips are the most common seat of extra-genital chancres in children.

By Professor Gaucher's courtesy I am able to quote his conclusions at length: "All observers are agreed in recognising the very great frequency of the site of the chancre on the face, and particularly of the chancre of the lips. As for chancre of the tonsil, of which Duncan Bulkley and Bœck have shown the extreme importance in children, we cannot support their opinion, as we have never seen primary tonsillar syphilis below the age of fifteen. It is fair to say that Bœck has observed it in Norway, and explains the frequency of tonsillar chancre in that country by the large number of poor families who share the same food utensils, and who possess only one spoon for a whole household. . . . We may conclude with the remark that just as there is no age protected from acquired syphilis, so there is no region of the skin or mucous membranes which may not at every age be the seat of syphilitic chancre, and where this may not be the result of some accidental contact."

### REFERENCES.

1 Colles' *Works*, New Syd. Soc. Ed., 1881, p. 276.
2 Diday, *Infantile Syphilis*, New Syd. Soc. Ed., 1859, *passim.*
3 *Ibid.*, 155.
4 *Ibid.*, 168.
5 Lancereaux, *Treatise on Syphilis*, New Syd. Soc. Ed., 1869, ii. 229.
6 *Ibid.*, ii. 232.
7 Sydenham, *Opera Omnia*, Syd. Soc. Ed., 1844, p. 309.
8 Lancereaux, *op. cit.*, ii. 237.
9 Schamberg, *J. Am. M. Ass.*, 1911, lvii. 783.

[10] Kehoe, *Lancet Clinic,* 1908, ciii. 8.

[11] Cullen, "Concerning Sibbens and the Scottish Yaws," *Caledonian M. J.*, 1911, viii. 336.

[12] Zarubin, "Ueber extra genitale syphilis infektion" (with bibliography), *Arch. f. Derm. u Syph.*, 1907, lxxxv. 293.

[13] Diday, *op. cit.*, 52.

[14] Allbutt, *System of Medicine,* vol. ii., pt. i., 1906, p. 718.

[15] Gottheil, *Progressive Medicine,* 1909, iii. 150.

[16] Gaucher and Flurin, "Sièges du Chancre Syphilitique chez les enfants au dessous de 15 ans," *Ann. des Mal. Veneri.*, 1910, 262.

